# Executive Functions’ Impact on Vocabulary and Verbal Fluency among Mono- and Bilingual Preschool-Aged Children

**DOI:** 10.11621/pir.2021.0405

**Published:** 2021-10-26

**Authors:** Maria S. Kovyazina, Ekaterina S. Oschepkova, Zlata V. Airapetyan, Maria K. Ivanova, Marfa I. Dedyukina, Margarita N. Gavrilova

**Affiliations:** a Lomonosov Moscow State University, Moscow, Russia; b Ammosov North-Eastern Federal University,Yakutsk, Russia

**Keywords:** Preschool age, speech, vocabulary, executive functions, working memory, inhibitory control, cognitive flexibility.

## Abstract

**Background:**

The phenomenon of multilingualism and its impact on child development are in the spotlight of latter-day psychology, and of utmost importance both for theory and practice. Language development is a strong predictor of psychological readiness for school and further academic success. At the same time, children’s mastery of written and oral speech in school education in a multilingual environment has several distinctive features. This study was dedicated to examining the influence of executive functions on the development of the vocabulary aspects of speech (both active and passive vocabulary) of mono- and bilingual children growing up in a bilingual environment.

**Objective:**

We aimed to analyze the relationship between bilingualism and language development (vocabulary and verbal fluency) and determine which executive functions may help overcome the resulting difficulties at preschool age.

**Design:**

Both monolingual and bilingual children participated in the study (n = 137 and n = 81, respectively). The children’s ages ranged from 6 to 7 years (M = 78.7 months, SD = 5.87). Two independent General Linear Models (GLM) were built to define which executive functions influenced the vocabulary and verbal fluency of the mono- and bilingual subjects (controlling for age, gender, and non-verbal intelligence as well).

**Results:**

The results confirmed that bilingualism is negatively related to language development, but showed that verbal working memory significantly helps bilinguals compensate for difficulties in developing vocabulary and verbal fluency.

**Conclusion:**

The study demonstrated that the ability to preserve and reproduce verbal information was of more significance for children’s vocabulary and verbal fluency than their language group (mono- or bilingual).

## Introduction

The phenomenon of multilingualism and its impact on child development are in the spotlight of latter-day psychology, and of utmost importance for both theory and practice. According to the definition given by the U.S. Department of Health and Human Services, 2008, a dual language learner is a person who acquires two or more languages simultaneously, *i.e*., learning a second language while still developing the first. Bi- or multilingual education and parenting is gaining popularity internationally and becoming more and more common. In many countries, children learn and use two or more languages from their early childhood.

The experience of parallel mastery of two languages to a certain extent affects the process of language mastery itself, and is tightly connected to cognitive systems of language application, reflection on the world, and self-regulation. The current study is dedicated to examining the impact of executive functions on the development of vocabulary aspects of speech (active and passive vocabulary) of mono- and bilingual children growing up in a bilingual environment.

### Language development

Language development is a complex phenomenon, defined by a whole number of mental processes related to children’s mastering of written and oral speech. Speech not only executes its main function of communication; it also provides for the development and functioning of other mental functions. Language development at preschool age has a significant impact on the development of cognitive skills (Diamond, Prevor, Callender, & Druin, 1997; Gooch, Thompson, Nash, Snowling, & Hulme, 2016; Pazeto, Seabra, & Dias, 2014; Rojas-Barahona et al., 2015) and emotional and personal development (Akhutina, Panikratova, Korneev, Matveeva, & Vlasova, 2019; Blair & Razza, 2007; Duff, Reen, Plunkett, & Nation, 2015; Slot, & von Suchodoletz, 2018; Weiland, Barata, & Yoshikawa, 2014). It also appears to be a strong predictor of psychological readiness for school learning (Pazeto et al., 2014; Rojas-Barahona et al., 2015; Japel, 2007).

A 2008 report by the U.S. National Institute for Literacy suggests distinguishing the following criteria when analyzing speech development: phonetic, vocabulary, syntactic, and symbolic. The phonetic criterion includes the perception of oral speech and independent sounds and words through the development of articulatory skills. The vocabulary aspect characterizes the success in the child’s mastery of words’ meanings (*i.e*., lexical items) and is reflected in the richness of the child’s vocabulary. Syntactic and grammar development is linked to the child’s mastery of language rules, necessary for constructing sentences. And last but not least, the symbolic aspect of language development covers the child’s mastery of graphic representations of sounds (letters) and the development of basic writing and reading skills.

### The Relationship between executive functions and language

The concept of executive functions has been actively explored in studies dedicated to a person’s self-regulation. We will be utilizing one of the most significant theoretical models of executive functions’ development, the one created by A. Miyake, Friedman, Emerson, Witzki, and Howerter (2000). Its advantage lies in its distinguishing several interconnected components of executive functions, which, if required by a corresponding research problem, can also be studied separately. According to this model, executive functions are a group of cognitive skills which provide for purposeful problem-solving and the ability to adapt to new situations. The authors suggest dividing executive functions into three main components: 1) working memory (visual and audial); 2) cognitive flexibility (allows the child to transition from one rule to another depending on the specifics of the situation); and 3) inhibitory control (allows the inhibition of impulsive reactions in favor of a voluntary and well-weighed answer).

At this moment, there are two main explanations of the nature of the relationship between language development and executive functions at preschool age. The first one derives from the idea of a gradual increase in the child’s cognitive ability due to the development of his/her executive functions (Gooch et al., 2016; Rojas-Barahona et al., 2015; Bierman, Nix, Greenberg, Blair & Domitrovich, 2008; Cain, Oakhill, & Bryant, 2004; Goff, Pratt, & Ong, 2005: Nilsen & Graham, 2009; Shaigerova, Shilko, & Zinchenko, 2019; Verhagen & Leseman, 2016; Weiland, Barata, & Yoshikawa, 2014).

For example, the development of working memory first allows the child to distinguish independent phonemes in the flow of verbal interaction, and remember them. It contributes to the growth of the number of lexical items he/she can use in further communication. The development of executive functions is viewed as the impetus for speech, as well as the intellectual, emotional, and personal development of a child. A whole range of experimental research dedicated to the purposeful development of executive functions, has revealed its connection to significant progress in children’s language development (Rojas-Barahona et al., 2015).

The second approach is based on the assumption that language development entails the development of executive functions (Botting et al., 2017; Bukhalenkova, Aslanova, Airapetyan, & Gavrilova, 2021; Henry, Messer, & Nash, 2012; Fuhs & Day, 2011; Lonigan, Wagner, Torgesen, & Rashotte, 2007). This explanation coheres with the principles of cultural-historical approach (Vygotsky, 2017), because the child’s inner speech, as understood by Lev Vygotsky, acts as the means of self-regulation. This inner speech serves the functions of the child’s goalsetting, planning, and performing of voluntary actions. This perspective has been supported by a number of research papers focused on the difficulties of attention and behavior control experienced by children with language development disorders (Henry, Messer, & Nash, 2012; Fuhs & Day, 2011; Petersen et al., 2013).

The relationship between executive functions and language was first registered in the lexical processing of the speech of monolingual subjects (Bohlmann et al., 2015; Fuhs & Day, 2011; Matthews et al., 2009). Moreover, Bierman and Weiland obtained evidence that the connection between those two domains didn’t just exist in one point in time. In fact, their relationship was a prolonged one, and the level of executive functions acted as a predictor for the development of children’s vocabulary (Bierman et al., 2008; Veraksa & Veraksa, 2021; Weiland et al., 2014).

For example, Weiland et al. discovered that the dynamic of vocabulary enrichment over one year in a kindergarten was determined by the child’s level of development of executive functions at the moment of admission (Weiland et al., 2014). Many experts point out that language skills evaluated at the beginning of preschool education, do not in fact influence either the means of development of executive functions or its pace. Equally, Bierman’s research (Bierman et al., 2008) demonstrated that all the individual components of executive functions (working memory, inhibitory control, and cognitive flexibility) forecast in large measure the growth of a child’s vocabulary.

Notwithstanding these results, two other major research papers revealed that there was indeed an association between lexical processing and executive functions, but a different one than was previously assumed (Oshchepkova, Bukhalenkova, & Almazova, 2021; Bohlmann et al., 2015; Vallotton & Ayoub, 2011). For example, Bohlmann’s longitudinal study found a bi-directional connection between the two aforesaid domains. The bigger the child’s vocabulary was at the beginning of the study, the better he/she solved the tasks that required additional skills, *i.e.,* the use of executive function throughout the research. Contrariwise, the better developed the child’s executive functions were *ad initium*, the more noticeable the dynamic of vocabulary growth was (Bohlmann et al., 2015).

Nevertheless, the study by Vallotton and Ayoub demonstrated that the size of a child’s vocabulary could be considered a reliable predictor of the development of his self-regulation, but not the other way around. The authors of the research emphasized that for children, language is a means of solving the self-regulation problem; it helps to focus their attention and thoughts (Vallotton & Ayoub, 2011). Research dedicated to the connection between vocabulary size and each separate component of executive functions, has led to the conclusion that it was the working memory which was related to the vocabulary size in the most consequent and powerful way (Cain et al., 2004; de Abreu et al., 2011; Morra & Camba, 2009; Verhagen & Leseman, 2016; Nilsen & Graham, 2009).

One should note that a similar positive connection was discovered on the morpho-syntactic level of processing; it was shown that children with poor working memory were facing more significant syntactic difficulties (Stanford & Delage, 2020; Delage & Frauenfelder, 2020). To conclude, our analysis of existing papers led us to assuming the presence of a bi-directional connection between the development of vocabulary and executive functions, where working memory plays a special role in the growth of vocabulary.

In the case of children who follow the typical developmental route, language and executive functions mutually impact and shape each other from the very first days of learning. Mastering a language creates a foundation for the development of a set of cognitive skills which support problem solving and adaptive behavior in new situations. Vygotsky’s assumption that inner speech contributes to the development of the child’s observation and regulation skills (including thinking and activity), was confirmed by research on children’s atypical speech development. Vygotsky suggested considering inner speech “not as speech minus sound, but as a speech function completely special and peculiar in its structure and mode of functioning, which /…/ is in an inextricable dynamic unity of transitions from one plan to another” (Vygotsky, 2017, p. 275). What he meant by this transition was that the organization of the child’s mental activity was in compliance with the situational context and assigned task. But then, various components of executive functions contribute to and support language development because: a) they help the child concentrate his/her attention on the child-adult interaction; b) provide for retaining visual and audial information (what the adult pronounces, and how); c) facilitate the formation of concepts (by establishing the relationship of the word and its meaning); and d) factor into the child’s control over his/her impulsive reactions and ability to act appropriately (finding the right words for a certain situation and making up his/her statements correctly).

It was to be expected that the development of bilingual children’s executive and language functions would have some sort of unique and complex dynamic by contrast with monolingual subjects. That would be explained by the fact that a bilingual child has to master two language systems simultaneously (for all processing levels, such as phonetical, lexical, and symbolical). Yet, there are clearly insufficient studies on the relationship between bilingual children’s language and executive functions, while there are quite a few for monolingual children ( Bialystok et al., 2005). Therefore, the goal of the present study was to analyze the relationship between bilingualism and language development (vocabulary and verbal fluency) and determine which executive functions may help overcome these children’s difficulties in language development at preschool age (considering also their age, gender and non-verbal intelligence level).

## Methods

### Participants

Two hundred and eighteen 6–7-year-old children participated in the current study (M = 78.7 months, SD = 5.87), 50.1% of them girls). All the subjects lived in the Republic of Sakha (Yakutia), and attended public kindergartens. The children were divided into groups (monolingual: n = 137, or bilingual: n = 81) in accordance with their preschool educators’ answers to the following questions: a) which language does the child speak most of the time in the classroom? and b) which language is used for child-parents communication? The results of the t-test for independence allowed us to be sure that the mono-and bilingual groups did not differ age-wise (p > 0.05). Assessment of the children’s executive functions was conducted individually by specially trained assistants. The assessment was performed in Russian in a separate quiet room provided by the kindergarten.

### Materials

#### Measures of language development

*The Peabody Picture Vocabulary Test 4th Edition* (PPVTTM-4) was used for the assessment of the subjects’ passive vocabulary, *i.e*., the volume of words that they could understand when perceived audibly (Dunn & Dunn, 2007). The latest edition of this test allows evaluation of a person’s active vocabulary (the volume of words that the child knows and is capable of naming without the interviewer’s assistance).

In order to evaluate the children’s verbal fluency, we applied the *Verbal Fluency Test (VFT)* (Akhutina, 2016). This tool is designed to measure the process of word and verbal fluency. A subject is given a minute and asked to name as many words as possible. The total score for Verbal Fluency is the number of productive associations, *i.e.,* all the words that were named without repetitions. The productivity score was calculated in the following way: every new word was assigned 1 point; the same applied to every combination of words; but if the child suggested combinations with a repeated word, each new one was assigned 0.5 point. If the subject produced a so-called “automatized sequence” (i*.e*., a learned and well-established sequence of words, such as Monday, Tuesday, Wednesday, etc.), the interviewer assigned 1 point for the whole row.

#### Measures of executive functions

*The Dimensional Change Card Sort (DCCS)* (Zelazo, 2006) is an executive functions-related task designed for the assessment of cognitive flexibility. The DCCS requires that the child sort cards; there are three rounds, and the rules change for each new one. First, sorting must be performed based on the color of the picture (pre-switch trial); then based on the shape (switch trial); and the last round combines contradictory rules— sorting should be based on either the color or the shape, depending on the presence of a frame in the picture (post-switch trial). For further analysis, we used the total score of this technique (the range went from 0 to 24 points).

The subtest *Inhibition* (Korkman, Kirk, & Kemp, 2007) is another technique aimed at assessing executive functions, in particular, children’s ability to inhibit automatized cognitive reactions. It consists of two series of shapes (squares, circles, and arrows). In the first stage the child is asked to name the shape or the direction in which the arrow is pointing (naming trial). In the second stage, the subject is supposed to name the shapes in a reverse manner, which means, say “a circle” when presented with a square, and vice versa. The directions of the arrows are also to be named conversely (inhibition trial). The total score range, used later in the analysis, went from 0 to 19.

Verbal working memory was measured by means of the subtest *Sentences Repetition* (Korkman et al., 2007). This tool consists of 17 sentences to be remembered, with their complexity gradually increasing throughout the test (the sentences get longer and more complex syntactically). For instance, if the first sentence contains only two words and is of the most basic structure (“Good night.”), sentence number 12 consists of 14 words, and is quite complicated structurally (“The woman, who stands next to a man in a green jacket, is my aunt.”). Leaving out a word or substituting a different word was categorized as an error, as well as a modification of word order or another change in the sentence structure. Four consecutive sentences with errors (0 points) meant failure, and the test was ended. We used the total scores (which ranged from 0 to 30) for the final analysis.

We used the subtest *Memory for Designs* (Korkman et al., 2007) to measure visual working memory. There were two parameters to consider: memorization of “images” (the task was to select some pictures matching an example, from a batch of similar pictures), and memorization of spatial locations of the pictures (children had to remember the exact position of the cards). Each successfully selected picture was assigned two points (“Content score”); one point was given for a correctly reproduced position (“Spatial score”). If the subject both correctly chose a picture and put it in the right place, he/she received two bonus points (“Bonus score”). Th us, there were four measurements available to rank the children’s visual working memory: a content score, a spatial score, a bonus score, and a total score (sum of all points obtained in all the tasks), in accordance with the NEPSY-II battery description.

#### Measure of non-verbal fluid intelligence

*The Russian adaptation of Raven’s Colored Progressive Matrices (CMPM)* (Rav en, Raven & Cort, 2002) was chosen for the assessment of non-verbal fluid intelligence. The respondents were asked to complete matrices of patterns and figures, choosing the right pattern among four options (only one could complete the matrix correctly). Points were accumulated until four consecutive mistakes were made. Then the trial was terminated. The total scores ranged from 0 to 36.

### Statistical Analysis

Descriptive statistics and Pearson’s r correlation were run for all the variables, to examine the data structure. For the main analysis, two independent General Linear Models (GLM) were built to define which executive functions influenced the vocabulary and verbal fluency of the mono- and bilingual subjects (controlling for age, gender, and non-verbal intelligence, as well). Jamovi software, version 1.0.7.0 (by The Jamovi Project) was used for the statistical analyses required for the current study.

## Results

### Descriptive statistics

See *Table 1* for descriptive statistics and Pearson’s r correlation for all study variables, including the subjects’ vocabulary, verbal fluency, non-verbal intelligence, and four components of executive functions.

**Table 1:** Descriptive statistics and for all study variables

	M	SD	1	2	3	4	5	6	7
1. Language group	1.38	0.486	–						
2. Non-verbal intelligence	26.41	9.150	0.037	–					
3. Vocabulary	107.96	33.983	–0.262**	0.113	–				
4. Verbal fluency	8.69	4.53	–0.222**	0.020	0.151	–			
5. Cognitive flexibility	19.15	2.734	0.113	0.105	0.162	0.230*	–		
6. Visual memory working	91.22	20.641	0.006	0.214*	0.152*	0.046	0.015	–	
7. Verbal memory working	16.89	4.962	–0.281***	0.026	0.387***	0.376***	–0.026	0.065	–
8. Inhibitory control	11.31	3.299	0.135*	0.229**	0.134*	0.109	0.078	0.175***	0.162**

Note. * Significant correlation at p < .05(2-tailed). ** Significant correlation at p < .01 (2-tailed). *** Significant correlation at p < .001 (2-tailed).

The outcome of Pearson’s r correlation analyses confirmed that bilingualism had a significantly negative relationship with the subjects’ vocabulary (r (217) = –.262, p < .01) and verbal fluency (r (217) = -.222, p < .01). It was positively related to their inhibitory control, though (r (217) = .135, p < .05). Apparently, respondents with a larger vocabulary performed better in all tasks focused on execution functions except cognitive flexibility (p > 0.05). Thus, they more often got higher scores on the visual working memory test (r (217) = .152, p < .01), the verbal working memory test (r (217) = .387, p < .001), and the inhibitory control test (r (217) = .134, p < .01). Children with well-developed verbal fluency often obtained higher scores for cognitive flexibility (r (217) = .230, p < .01), and verbal working memory (r (217) = .376, p < .001). Assessment results for executive functions and speech development are detailed in *Table 2* separately for mono- and bilingual children.

**Table 2:** Results of a complex study assessment separately for mono- and bilingual children

	Language Group	M	SD	Median	Min	Max
Non-verbal Intelligence	Bilingual	28.79	10.34	33	1	36
	Monolingual	28.66	7.20	31	6	36
Vocabulary	Bilingual	106.33	20.87	107	63	147
	Monolingual	121.39	33.21	118.00	10	228
Verbal Fluency	Bilingual	8.28	4.03	8.00	1	16
	Monolingual	10.14	4.77	10	1	26
Verbal Fluency	Bilingual	8.28	4.03	8.00	1	16
	Monolingual	10.14	4.77	10	1	26
Cognitive Flexibility	Bilingual	19.63	2.43	20	14	24
	Monolingual	19.05	2.56	19.00	11	24
Visual Working Memory	Bilingual	89.48	20.24	86	50	120
	Monolingual	89.21	19.97	91.00	53	120
Verbal Working Memory	Bilingual	16.11	4.15	17.00	1	23
	Monolingual	18.06	5.22	19	3	29
Inhibition Combined	Bilingual	11.94	3.00	12.00	6	19
	Monolingual	11.01	3.56	11	3	19

### Vocabulary and verbal fluency

We built two independent General Linear Models (GLM) with the purpose of examining what levels of vocabulary and verbal fluency (dependent variable) were demonstrated by the subjects, depending upon their executive functions (independent variable). The following factors were controlled as well: language group (mono- and bilinguals), age (continuous in months), gender (categorical with two levels), and non-verbal intelligence (continuous).

### Vocabulary

One GLM (Vocabulary (PPVT-4) ~ 1 + `Language group` + Gender + `Age` + `Non-verbal Intelligence` + `Cognitive Flexibility` + `Visual Working Memory` + `Inhibition Combined` + `Verbal Working Memory`) was built to find out which executive functions influenced the subjects’ vocabulary (age, gender, and non-verbal intelligence controlled). [Model with Adj. R-squared = 0.506.] An ANOVA Omnibus test revealed that the model described the data correctly: F (8) = 5.369, p < .001, ɳ^2^p = .506. According to Fixed Effects Parameter Estimates, only verbal working memory had a significant effect on the children’s vocabulary: F (1) = 9.366, p = .004, ɳ^2^p = .182. No significant effects of language group, gender, age, non-verbal intelligence, cognitive flexibility, visual working memory, and inhibition were registered. The vocabulary test score of the respondents from both language groups (mono-and bilinguals) was higher in correspondence with the development of their verbal working memory.

#### Verbal Fluency

Then we built a GLM (Verbal Fluency ~ 1 + ` Language group` + Gender + `Age` + `Non-verbal Intelligence` + `Cognitive Flexibility` + `Visual Working Memory` + `Inhibition Combined` + `Naming Combined` + `Verbal Working Memory`), to see which executive functions affected the subjects’ verbal fluency. [Model with Adj. R-squared = 0.316.] An ANOVA Omnibus test indicated that the model described the data well: F (9) = 1.59, p < .0161, ɳp = .316. According to Fixed Effects Parameter Estimates, only verbal working memory had a significant effect on the children’s verbal fluency: F (1) = 5.975, p = .020, ɳ^2^p = .116. Language group, age, gender, and nonverbal intelligence were controlled. No significant effects of language group, gender, age, non-verbal intelligence, cognitive flexibility, visual working memory, and inhibition were discovered.

## Discussion

The key goal set for our study was to analyze the relationship between bilingualism and language development (vocabulary and verbal fluency) and determine which executive functions may help overcome difficulties in language development at preschool age, while controlling for age, gender, and non-verbal intelligence. First, we explored the relationship among vocabulary, verbal fluency, executive functions, and bilingualism. It was revealed that bilingualism had a significant negative correlation with the subjects’ inhibitory control, and a positive correlation with their inhibitory control. Vocabulary size turned out to be related positively to all executive functions except cognitive flexibility.

At the next stage, we studied the influence of executive functions on vocabulary and verbal fluency of bi- and monolingual children (age, gender, and non-verbal intelligence controlled). The outcome of that analysis proved that the size of vocabulary and the level of verbal fluency are determined to a major extent by verbal working memory. As per our study results, other executive functions do not have any significant effect on the vocabulary and verbal fluency of bi- and monolingual children (language group, age, gender, and non-verbal intelligence controlled). Besides, the language group (mono-/bilingual) was of no significance in predicting vocabulary size and verbal fluency, if the models included verbal working memory.

The positive impact of verbal working memory on verbal fluency among bi- and monolingual children could possibly be understood as linked to the growth of the child’s cognitive abilities due to the development of his or her capability to remember verbal information (Gooch et a l., 2016; Rojas-Bara hona et al., 2015; Weiland et al, 2014; Bierman, 2008; Cain, 2004; Goff, 2005; Nilsen, 2009; Verhagen, 2016). It has been previously demonstrated on a monolingual sample that working memory development allows the child to first distinguish independent phonemes in the flow of verbal interaction, and remember them. This contributes to the growth in the number of the child’s lexical items. Our study results indicate that this pattern can be also observed when a child uses two languages simultaneously. Verbal working memory indeed can be considered one of the impetuses of a child’s verbal development (Rojas-Barahona et al., 2015).

Nevertheless, we should not dismiss the idea that the development of vocabulary and verbal fluency can also result in the development of the child’s executive functions (Botting et al., 2017; Henry, 2012; Fuhs, 2011; Lonigan, 2007). As Vygotsky noted, egocentric speech “…becomes a means of thinking in the proper sense, *i.e*., it begins to fulfill the function of forming a plan for solving the problem arising in behavior” (Vygotsky, 1982, V. 2, p. 49). Th erefore, a rich vocabulary and/or verbal fluency can open new opportunities for child’s self-regulation. When it comes to current study results, we can assume that aforementioned rich vocabulary and/or verbal fluency allows the child to recognize, understand, and remember a larger quantity of verbal information.

## Limitations

The results we obtained should be interpreted in the context of certain limitations. To start with, the entire sample came from the same region (the Republic of Sakha Yakutia). If we desire further research to replicate these results, a more diverse sample will be needed, which will be more representative. Another limitation was related to the age range of the respondents (only 6–7-year-olds participated in the study). This, unfortunately, was due to the constraints of existing organizational resources. If we want to explore the age dynamic in relation to vocabulary and verbal fluency, and its association with the regulatory functioning of mono- and bilingual children, a new study with a broader age range of the sample is required.

Yet, despite the indicated limitations, this study provides an important contribution to the research field focused on language development of bilingual children. This is due to the evidence we obtained of the positive effect of verbal working memory on children’s vocabulary and verbal fluency (age, gender and non-verbal intelligence controlled).

## Conclusion

This study’s results confirmed that bilingualism in preschool years is negatively related to language development and demonstrated that the ability to preserve and reproduce verbal information is more significant for children’s vocabulary and verbal fluency than the child’s language group. The results obtained may be helpful in the elaboration of programs aimed at reducing learning difficulties for bilingual children.
